# The risks of hepatocellular carcinoma development after HCV eradication are similar between patients treated with peg-interferon plus ribavirin and direct-acting antiviral therapy

**DOI:** 10.1371/journal.pone.0182710

**Published:** 2017-08-10

**Authors:** Yuko Nagaoki, Michio Imamura, Hiroshi Aikata, Kana Daijo, Yuji Teraoka, Fumi Honda, Yuki Nakamura, Masahiro Hatooka, Reona Morio, Kei Morio, Hiromi Kan, Hatsue Fujino, Tomoki Kobayashi, Keiichi Masaki, Atsushi Ono, Takashi Nakahara, Tomokazu Kawaoka, Masataka Tsuge, Akira Hiramatsu, Yoshiiku Kawakami, C. Nelson Hayes, Daiki Miki, Hidenori Ochi, Kazuaki Chayama

**Affiliations:** 1 Department of Gastroenterology and Metabolism, Applied Life Science, Institute of Biomedical & Health Science, Hiroshima University, Hiroshima, Japan; 2 Liver Research Project Center, Hiroshima University, Hiroshima, Japan; 3 Natural Science Center for Basic Research and Development, Hiroshima University, Hiroshima, Japan; 4 Laboratory for Digestive Diseases, RIKEN Center for Integrative Medical Sciences, Hiroshima, Japan; Kaohsiung Medical University Chung Ho Memorial Hospital, TAIWAN

## Abstract

The risk of hepatocellular carcinoma (HCC) development is reduced following viral elimination by interferon therapy in chronic hepatitis C patients. However, the risk in patients treated with interferon-free direct-acting antivirals (DAAs) is unknown. We evaluated chronic hepatitis C patients who achieved viral eradication by pegylated-interferon plus ribavirin (PEG-IFN/RBV, n = 244) or daclatasvir plus asunaprevir (DCV/ASV, n = 154) therapy. None of the patients had prior history of HCC or antiviral therapy. The median observation period after the end of treatment for the PEG-IFN/RBV and DCV/ASV groups were 96 (range 10–196) and 23 (range 4–78) months, respectively. During the observation period, HCC developed in 13 (5.3%) and 7 (4.5%) patients in the PEG-IFN/RBV and DCV/ASV groups, respectively. The cumulative HCC development rate after 1-, 3- and 5-years (0.4%, 3% and 5% for the PEG-IFN/RBV group and 0.6%, 9% and 9% for the DAA group, respectively) were similar between the two groups. Propensity score matching analysis also showed no significant difference in HCC development rates between the two groups. Serum AFP levels decreased to similar levels between PEG-IFN/RBV and DCV/ASV groups following the achievement of viral eradication. The risk for HCC development following viral eradication by IFN-free DAA therapy may be similar to that in IFN-based therapy.

## Introduction

Chronic hepatitis C virus (HCV) infection is a leading cause of cirrhosis, liver failure, and hepatocellular carcinoma (HCC) [1, 2]. Particularly among cirrhotic patients with HCV infection, the HCC incidence rate steadily increases, with a yearly reported incidence of 1.4% to 7% [3, 4]. The goal of chronic HCV infection treatment is eradication of the virus in order to prevent HCC development. Interferon (IFN) has long been used for anti-HCV therapy, and achievement of sustained virological response (SVR) by peg-interferon plus ribavirin (PEG-IFN/RBV) therapy could reduce the incidence of HCC development in patients with chronic HCV infection by reducing liver inflammation, fibrosis, and serum alanine aminotransferase (ALT) levels [5–10].

Recently, direct-acting antivirals (DAAs) that selectively inhibit HCV proteins, such as nonstructural protein (NS)3/4A protease, NS5A, and NS5B polymerase, have been approved for treatment of chronic hepatitis C patients in many parts of the world. In Japan, an IFN-free DAA therapy with daclatasvir, an NS5A replication complex inhibitor, and asunaprevir, an NS3/4A protease inhibitor, was first approved for treatment of genotype 1 HCV-infected patients in 2014 [11]. Real-world study results from daclatasvir plus asunaprevir (DCV/ASV) therapy showed an approximately 95% SVR rate [12]. Subsequently, new DAA combination therapies such as sofosbuvir plus ledipasvir [13] ombitasvir/paritaprevir/ritonavir [14,15] and elbasvir plus grazoprevir have also recently been approved in Japan.

Similar to PEG-IFN/RBV therapy, achievement of SVR by IFN-free DCV/ASV therapy has been shown to result in reduction of liver fibrosis markers and serum alfa-fetoprotein (AFP) levels [16]. A recent report showed a reduction in the incidence [17,18] and the occurrence [19] of HCC after HCV eradication in patients treated with DAA therapy. However, it is not known whether or not IFN-free DCV/ASV therapy reduces the risk of HCC development to the same extent as PEG-IFN/RBV therapy. In this study, we compared the risks of HCC development in patients who achieved SVR by DAA treatment to that in patients treated by PEG-IFN/RBV therapy.

## Materials and methods

### Patients

We reviewed 398 consecutive patients with genotype 1 HCV infection who achieved viral eradication by antiviral therapy at Hiroshima University Hospital between January 1995 and April 2015. The PEG-IFN/RBV group included 244 consecutive patients who achieved viral eradication with PEG-IFN/RBV for 48 to 72 weeks between 1995 and 2013. The standard treatment was as follows: PEG-IFNα2b (PEG-Intron, MSD, Tokyo, Japan) was injected subcutaneously at a median dose of 1.5 μg/kg once per week. RBV (Rebetol, MSD) was administered after breakfast and dinner. The RBV dose was adjusted by body weight (600 mg for <60 kg; 800 mg for 60–80 kg; and 1,000 mg for >80 kg). Twenty-six out of 154 patients in the DCV/ASV group participated in phase 2 and 3 clinical trials (clinicaltrials.gov identifier NCT01051414 and NCT01497834, respectively) conducted between 2010 and 2012 in which they received 60 mg of daclatasvir plus 200–600 mg BID of asunaprevir daily for 24 weeks. The remaining 128 patients received 24 weeks of treatment with 60 mg once daily of daclatasvir (Daklinza, Bristol-Myers, NY) and 200 mg twice daily of asunaprevir (Sunvepra, Bristol-Myers) between 2014 and 2015.

Patients with prior history of HCC before beginning antiviral therapy were excluded from the study. All subjects gave written informed consent to participate in the study according to the process approved by the ethical committee of Hiroshima University Hospital and conforming to the ethical guidelines of the 1975 Declaration of Helsinki.

### Clinical and laboratory assessments

Clinical and laboratory assessments were performed before treatment. HCV RNA levels were measured using COBAS 135 TaqMan HCV test (Roche Diagnostics, Tokyo, Japan). The detection limit for the assay was 1.2 log IU/mL. HCV genotype was determined by sequence determination of the 5' non-structural region of the HCV genome, followed by phylogenetic analysis. FIB4 index was calculated as a surrogate marker of liver fibrosis [19]. FIB4 index = age (years) × aspartate aminotransferase (AST) [IU/L] / (platelet count [10^9^/l] × (ALT [IU/L])^1/2^). According to Sterling et al [20], we considered a FIB4 index of ≥3.25 as severe fibrosis.

### HCC surveillance

All patients underwent HCC surveillance using tumor markers, ultrasonography, and/or dynamic computed tomography at least biannually after HCV eradication. The diagnosis of HCC was based on the hypervascular staining pattern of the arterial phase and the hypovascular staining pattern of the portal phase, and was confirmed by dynamic CT, magnetic resonance imaging, and/or angiography. Tumors without enhancement upon imaging were diagnosed by fine-needle biopsy.

### Single-nucleotide polymorphism (SNP) genotyping

We genotyped each patient for two SNPs: rs8099917 in the *IL28B* locus, which is associated with response to IFN therapy [21, 22]; and rs1012068 in the *DEPDC5* locus, which is associated with the risk of HCC development in chronic HCV-infected Japanese patients [23]. Samples were genotyped using the Invader assay, as described previously [24].

### Statistical analysis

Continuous variables were analyzed using the Mann-Whitney *U*-test. Categorical variables were compared using the chi-square or Fisher exact test, as appropriate. The incidence of HCC was calculated by the Kaplan-Meier method, and differences between groups were assessed by the log-rank test. Multivariate analysis was conducted with a Cox proportional hazard model using the stepwise selection of variables or with two logistic analyses. All statistical analyses were performed using the SPSS software package (version SPSS 23.0, IBM, Armonk, NY, USA). A *P* value <0.05 was considered statistically significant.

## Results

### Characteristics of patients

The clinical characteristics of the 398 patients are shown in Table 1. Patients in the DAA group were older (median age 73 vs. 59 years), mainly female (62.3% vs. 52.2%), had lower platelet counts, leukocyte counts and hemoglobin levels, and had lower serum ALT and albumin levels than patients in the PEG-IFN/RBV group. HbA1c levels and history of diabetes mellitus were significantly higher, and histories of alcohol intake, hypertension and hyperlipidemia were also higher in the DCV/ASV group compared with the PEG-IFN/RBV group. HCV-RNA level was higher in the DCV/ASV group compared with the PEG-IFN/RBV group. AFP levels were higher, and liver fibrosis was more severe in the DAA group compared with the PEG-IFN/RBV group.

**Table 1:** Clinical characteristics of 398 patients.

	PEG-IFN/RBV (n = 244)	DCV/ASV (n = 154)	*P* value
Age at HCV eradication (years)	59 (22–77)	73 (37–90)	<0.001
Gender (male/female)	127/117	58/96	0.005
Aspartate aminotransferase (IU/L)	40 (16–587)	39 (12–272)	0.467
Alanine aminotransferase ALT (IU/L)	48 (20–878)	44 (15–339)	<0.001
Albumin (g/dL)	4.4 (1.3–5.5)	4.0 (2.3–4.9)	<0.001
γ-glutamyl transpeptidase (IU/L)	33 (8–508)	32 (11–307)	0.247
Platelet count (×10^4^/μL)	15.9 (4.6–75.9)	13.9 (3.4–82.2)	0.004
Leukocyte count (×10^4^/μL)	4950 (1680–9660)	4490 (1380–12400)	0.029
Hemoglobin (g/dlL)	13.9 (6.3–17.8)	12.9 (8.3–17.3)	0.001
Total cholesterol (mg/dL)	175 (100–274)	168 (79–265)	0.020
Triglyceride (mg/dL)	94 (11–404)	125 (35–517)	0.144
HbA1c (%)	5.2 (4.0–8.2)	5.9 (4.2–9.4)	0.001
Alfa-fetoprotein (ng/mL)	9.1 (1.6–87.2)	16.9 (1.2–343)	<0.001
Body mass index (kg/m^2^)	22.2 (16.1–33.9)	22.7 (14.7–39.4)	0.524
Alcohol intake (yes/no)	67/175	26/116	0.016
Hypertension (yes/no)	62/182	82/68	0.001
Diabetes mellitus (yes/no)	29/215	36/114	0.003
Hyperlipidemia (yes/no)	34/210	44/106	0.001
FIB4 index	2.25(0.32–22.459)	3.41(0.43–20.108)	<0.001
FIB4 index (<3.25/3.25≤)	175/69	74/84	<0.001
HCV RNA (log/IU/mL)	4.7 (1.2–8.7)	6.0(0.8–7.6)	<0.001
*IL28B* genotype rs8099917 (TT/TG+GG)	199/45	91/63	<0.001
*DEPDC5* genotype rs1012068 (TT/TG+GG)	185/59	122/32	0.463

Categorical data are represented as numbers of patients, and continuous data is represented as median and range.

PEG-IFN/RBV, peg-interferon plus ribavirin; DCV/ASV, daclatasvir plus asunaprevir; alcohol intake, ≥80 g/day for more than 5 years

### HCC development rate

**Fig 1:**
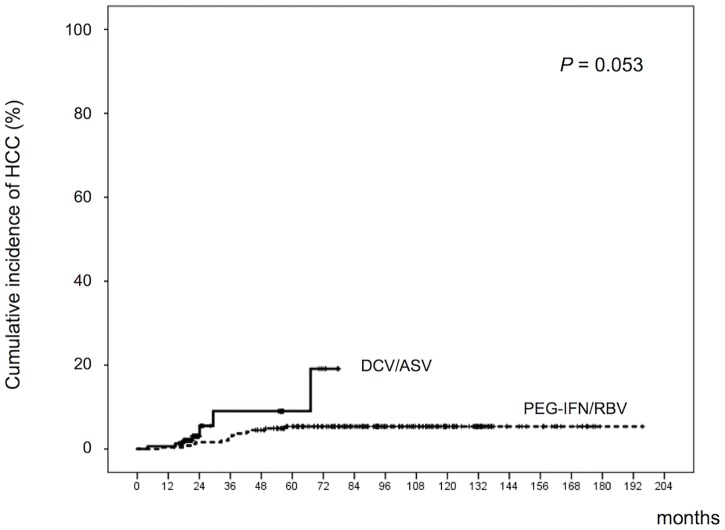
Cumulative hepatocellular carcinoma (HCC) development. Patients who achieved viral eradication by peg-interferon and ribavirin (PEG-IFN/RBV) or daclatasvir plus asunaprevir (DCV/ASV) therapies were analyzed.

The median observation period after the end of treatment for the PEG-IFN/RBV and DCV/ASV groups were 96 (range 10–196) and 23 (range 4–78) months, respectively. During the observation period, HCC developed in 13 out of 244 (5.3%) patients in the PEG-IFN/RBV group and 7 out of 154 (4.5%) patients in the DCV/ASV group. The median period from the end of treatment to diagnosis of HCC was 35 (range 10–57) months in the PEG-IFN/RBV group and 22 (range 4–67) months in the DCV/ASV groups (*P* = 0.54). The cumulative HCC development rates at 1, 3 and 5 years were 0.4%, 3% and 5% for the PEG-IFN/RBV group and 0.6%, 9% and 9% for the DCV/ASV group, respectively (*P* = 0.053) (Fig 1).

### Propensity score matching analysis

**Fig 2:**
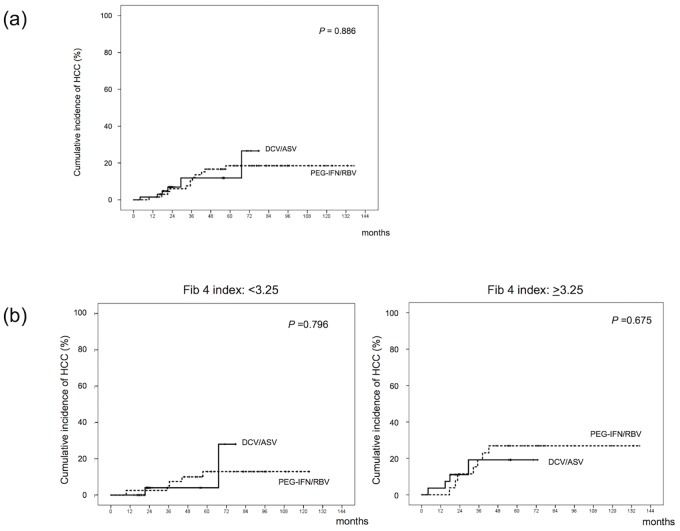
Cumulative hepatocellular carcinoma (HCC) development in propensity score matched patients. (a) 66 propensity score-matched patients in each of peg-interferon and ribavirin (PEG-IFN/RBV) and daclatasvir plus asunaprevir (DCV/ASV) groups were analyzed. (b) Patients were grouped by FIB4 index.

To overcome bias due to differences in the distributions of covariates among patients treated with PEG-IFN/RBV or DCV/ASV, one-to-one matches were created using propensity score analysis [25, 26]. Variables entered in the propensity model included age, gender, platelet counts, leukocyte counts, hemoglobin levels, serum albumin and ALT levels, HbA1c, histories of diabetes mellitus and alcohol intake, hypertension, and hyperlipidemia. The model was then used to obtain a one to-one match by using the nearest-neighbor matching method [27, 28] in 244 PEG-IFN/RBV-treated and 154 DCV/ASV-treated patients, resulting in a sample size of 66 patients per cohort (Table 2). The cumulative HCC development rate at 1-, 3- and 5-year were 1.5%, 10% and 19% for the PEG-IFN/RBV group and 1.5%, 10% and 12% for the DCV/ASV group, respectively (*P* = 0.886) (Fig 2A). Propensity score matching analysis also showed similar HCC development rates in the two groups.

**Table 2:** Characteristics of propensity score-matched patient treated with PEG-IFN plus RBV or daclatasvir plus asunaprevir.

	PEG-IFN/RBV (n = 66)	DCV/ASV (n = 66)	*P* value
Age at HCV eradication (years)	65 (47–77)	65 (37–88)	N.S.
Gender (male/female)	29/37	32/34	N.S.
Aspartate aminotransferase (IU/L)	38 (16–209)	39 (13–272)	N.S.
Alanine aminotransferase (IU/L)	36 (13–228)	37 (11–339)	N.S.
Albumin (g/dL)	4.3 (1.3–5.5)	4.2 (2.3–4.7)	N.S.
γ-glutamyl transpeptidase (IU/L)	32 (12–285)	37 (11–307)	N.S.
Platelet count (×10^4^/μL)	14.7 (4.5–75.9)	14.3 (3.4–82.0)	N.S.
Leukocyte count (×10^4^/μL)	4995 (1810–9340)	4875 (2090–9400)	N.S.
Hemoglobin (g/dL)	13.6 (10.0–17.3)	13.7 (10.4–17.2)	N.S.
Total cholesterol (mg/dL)	174 (104–274)	175 (108–275)	N.S.
Triglyceride (mg/dL)	98 (39–404)	110 (41–517)	N.S.
HbA1c (%)	5.2 (4.7–7.3)	5.8 (4.6–8.6)	N.S.
Alfa-fetoprotein (ng/mL)	5.6 (2.6–87.2)	5.9 (1.2–220)	N.S.
Body mass index (kg/m^2^)	22.3 (17.6–25.6)	23.1 (15.8–39.4)	N.S.
Alcohol intake (yes/no)	16/50	17/49	N.S.
Hypertension (yes/no)	23/43	31/35	N.S.
Diabetes mellitus (yes/no)	20/46	21/45	N.S.
Hyperlipidemia (yes/no)	18/48	23/43	N.S.
FIB4 index (<3.25/3.25≤)	33/23	39/27	N.S.
FIB4 index	2.8(1.47–18.2)	2.7(0.43–20.11)	N.S.
HCV RNA (log/IU/mL)	6.4 (1.3–7.3)	6.1 (1.8–7.6)	N.S.
*IL28B* rs8099917 (TT/TG+GG)	39/27	32/34	N.S.
*DEPDC5* rs1012068 (TT/TG+GG)	44/22	52/14	N.S.

Categorical data are represented as numbers of patients, and continuous data is represented as median and range.

PEG-IFN/RBV, peg-interferon plus ribavirin; DCV/ASV, daclatasvir plus asunaprevir; N.S., not significant; alcohol intake, ≥80 g/day for more than 5 years

To compare the HCC development rate between PEG-IFN/RBV- and DCV/ASV-treated patients according to liver fibrosis, patients were grouped by FIB4 index. Cumulative HCC development rates were similar between PEG-IFN/RBV and DCV/ASV groups both in patients with FIB4 index of <3.25 (*P* = 0.796) and ≥3.25 (*P* = 0.675) (Fig 2B).

### Risk factors for the development of HCC in patients treated with DAAs

Risk factors for the development of HCC in patients who achieved viral eradication by DCV/ASV were analyzed. Univariate analysis showed that gender (*P* = 0.019) and serum AFP levels (*P* = 0.046) were significantly correlated with HCC development after HCV eradication. However, no factor was identified as an independent risk factor for HCC development. The SNPs within *IL28B* and *DEPDC5* were also not associated with HCC development.

### AFP levels before and after antiviral therapy

**Fig 3:**
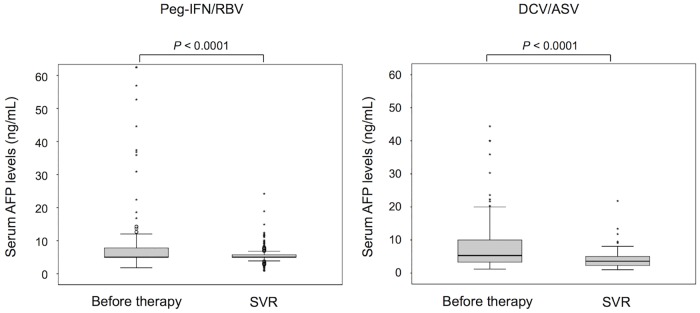
Sequential changes of serum alfa-fetoprotein (AFP). Serum AFP levels before therapy and six months after the end of the treatment in patients treated with peg-interferon plus ribavirin (PEG-IFN/RBV) or daclatasvir plus asunaprevir (DCV/ASV). In these box-and-whisker plots, lines within the boxes represent median values; the upper and lower lines of the boxes represent the 75th and 25th percentiles, respectively; the upper and lower bars outside the boxes represent the 90th and 10th percentiles, respectively.

Serum AFP levels are associated with hepatocarcinogenesis; therefore, sequential changes in serum AFP levels were analyzed in patients without HCC development. The levels decreased significantly and to similar levels following achievement of HCV eradication both in patients treated with PEG-IFN/RBV and DCV/ASV (Fig 3).

### HCC development rate according to the timing of DCV/ASV therapy

In the present study, 26 out of 154 patients in the DCV/ASV group were treated between 2010 and 2012, and the remaining 128 patients were treated between 2014 and 2015. The median observation period after the end of treatment for each groups were 56 (range 29–78) and 22 (range 4–28) months, respectively. We analyzed HCC development rates in each group. Propensity score matching resulted in the selection of 18 patients in each of DCV/ASV between 2010 and 2012 and PEG-IFN/RBV groups, and 61 patients in each of DCV/ASV between 2014 and 2015 and PEG-IFN/RBV groups, respectively (S1 and S2 Tables). The cumulative HCC development rates were similar to PEG-IFN/RBV-treated patients in DCV/ASV-treated patients both in between 2010 and 2012 (*P* = 0.616) and between 2014 and 2015 (*P* = 0.525) groups (S1 Fig).

## Discussion

This study examined the cumulative incidence of HCC development after HCV eradication by PEG-IFN/RBV or DCV/ASV treatment. During the follow-up period, HCC developed in 13 out of 244 (5.3%) of the PEG-IFN/RBV group and 7 out of 154 (4.5%) of the DCV/ASV group, and the cumulative HCC development rate tended to be high in DCV/ASV groups (*P* = 0.053). To overcome bias due to the different distributions of covariates among patients treated with PEG-IFN/RBV or DCV/ASV, one-to-one matches were created using propensity score analysis. The cumulative HCC development rate after 1-, 3- and 5-years were 1.5%, 10% and 19% for the PEG-IFN/RBV group and 1.5%, 10% and 12% for the DCV/ASV group, respectively. Propensity score matching analysis also showed similar rates of HCC development in the two groups. Based on the results of this analysis, one can argue that the risk of HCC development after HCV eradication achieved by DCV/ASV is similar to that of IFN therapy. In the present study, HCC development risk was similar between the PEG-IFN/RBV and the DCV/ASV groups in both patients with and without advanced liver fibrosis. In contrast, Conti, et al. reported that virus eradication by DAAs treatment did not reduce occurrence of HCC development in HCV-infected cirrhosis patients [29]. Further analysis is needed to clarify the relationship between HCC development risk after HCV eradication by DAAs treatment and liver fibrosis.

A recent study showed a high rate and unexpected pattern of HCC recurrence after HCV eradication by IFN-free DAA therapy in patients with HCV-related HCC [30]. Although the mechanism underlying this unexpected early HCC recurrence is unknown, it is possible that HCV eradication by DAA therapy could enhance HCC development or recurrence in patients who have elevated risk for HCC. However, the present study showed no evidence for an increase in HCC development following achievement of HCV eradication by DAA therapy.

The present study has several limitations. First, the observation period was relatively short in the DCV/ASV group; the median observation period was only 23 months in this group compared with 96 months in the PEG-IFN/RBV group. Furthermore, only 7 patients developed HCC in DCV/ASV group. This short observation period and the small number of patients who developed HCC might underlie the lack of significance between the two groups.

Second, patients who were treated with PEG-IFN/RBV and those treated with DCV/ASV differ with respect to many host and viral factors that potentially affect HCC development. Although propensity score analysis showed a similar HCC development risk between the two groups, the propensity score matching may not completely compensate for these differences among the patient populations. Large-scale, long-term follow-up studies that include patients treated with other DAA regimens, such as sofosbuvir plus ledipasvir, ombitasvir/paritaprevir/ritonavir, and elbasvir plus grazoprevir, should be performed.

Previous studies have shown that advanced liver fibrosis, male gender, older age, high AFP levels, greater alcohol intake, complications from diabetes mellitus, and obesity were risk factors for HCC development after HCV eradication by IFN treatment [17,18, 31–34]. The present study showed that male gender and AFP levels ≥10 ng/mL were associated with HCC development after HCV eradication by DCV/ASV therapy; however, no factor was identified for independent risk for HCC development. The small number of patients who developed HCC might be associated with the absence of independent factors. AFP is a surrogate marker for risk of HCC development. Previous reports showed that AFP levels after the completion of IFN treatment were useful predictors for HCC development in chronic hepatitis C patients who achieved viral eradication [10, 34]. In the present study, serum AFP levels decreased to similar levels following achievement of SVR both in the PEG-IFN/RBV and the DCV/ASV groups, suggesting the possibility of reduced potential for HCC development. Future analysis to identify predictors for HCC development after HCV eradication to examine whether AFP levels could be a surrogate marker for HCC development in patients who achieved SVR by IFN-free DAA therapy using a larger number of patients is needed.

Previous studies showed that *IL28B* and *DEPDC5* genotypes were strongly associated with IFN treatment response and HCC development in chronic hepatitis C patients, respectively [21–23]. However, the present study showed that *IL28B* and *DEPDC5* genotypes were not independent risk factors for HCC after HCV eradication. Recent genome-wide association study identified the association between the SNP rs17047200 in the *TLL1* locus and HCC development after HCV eradication in patients treated with IFN-based treatments [35]. Future analysis is expected to identify SNPs associated with HCC development after HCV eradication, particularly in patients treated with DAAs.

In conclusion, we demonstrated that the risk of HCC development in patients infected with HCV genotype 1 after achieving viral eradication with DAA therapy is similar to that for PEG-IFN/RBV therapy.

## Supporting information

S1 Fig Cumulative hepatocellular carcinoma (HCC) development in propensity score matched patients. Patients treated with peg-interferon and ribavirin (PEG-IFN/RBV) or daclatasvir plus asunaprevir (DCV/ASV) were analyzed. Patients were grouped by the timing of DCV/ASV treatment.
(TIFF)

S1 Table Characteristics of propensity score-matched patient treated with PEG-IFN plus RBV or daclatasvir plus asunaprevir between 2010 and 2012. (DOCX)

S2 Table Characteristics of propensity score-matched patient treated with PEG-IFN plus RBV or daclatasvir plus asunaprevir between 2014 and 2015. (DOCX)

## Abbreviations

AFP: alfa-fetoprotein
ALT: alanine aminotransferase
AST: aspartate aminotransferase
DAA: direct-acting antiviral
DCV/ASV: daclatasvir plus asunaprevir
HCC: hepatocellular carcinoma
HCV: hepatitis C virus
HR: hazard ratio
IFN: interferon
NS: nonstructural protein
PEG-IFN/RBV: pegylated-interferon plus ribavirin
SVR: sustained virological response
