# Supplementary figures and images for: The risks of hepatocellular carcinoma development after HCV eradication are similar between patients treated with peg-interferon plus ribavirin and direct-acting antiviral therapy
Source: PLoS One. 2017 Aug 10;12(8):e0182710. doi: 10.1371/journal.pone.0182710 (PMC5552231; doi:10.1371/journal.pone.0182710)

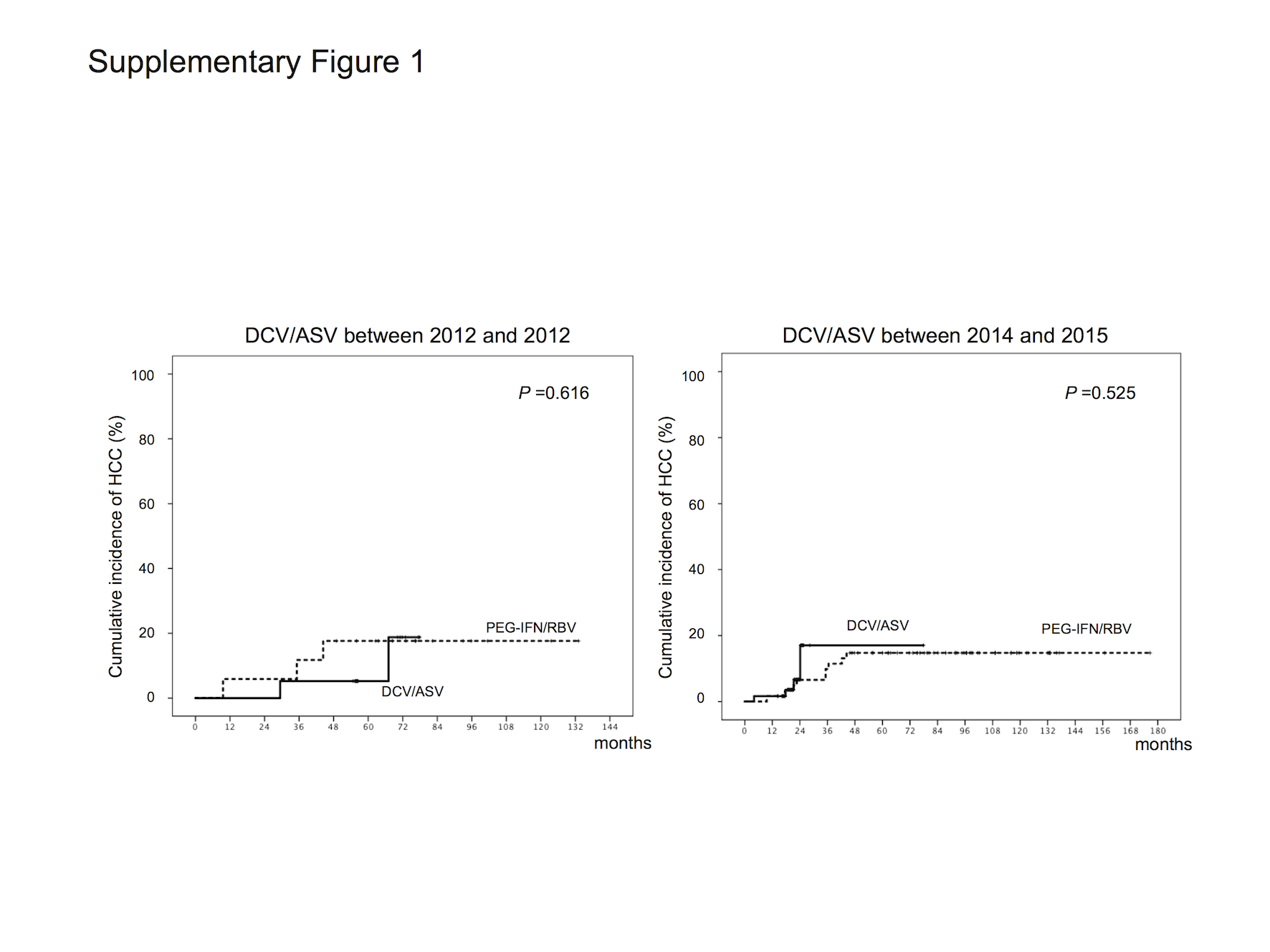

Supplement: S1 Fig — Patients treated with peg-interferon and ribavirin (PEG-IFN/RBV) or daclatasvir plus asunaprevir (DCV/ASV) were analyzed. Patients were grouped by the timing of DCV/ASV treatment. (TIFF) [file pone.0182710.s001.tiff]
