# Supplementary material for: The risks of hepatocellular carcinoma development after HCV eradication are similar between patients treated with peg-interferon plus ribavirin and direct-acting antiviral therapy
Source: PLoS One. 2017 Aug 10;12(8):e0182710. doi: 10.1371/journal.pone.0182710 (PMC5552231; doi:10.1371/journal.pone.0182710)
Supplement: S1 Table — (DOCX) [file pone.0182710.s002.docx]

**Supporting table 1.** Characteristics of propensity score-matched patient treated with PEG-IFN plus RBV or daclatasvir plus asunaprevir between 2010 and 2012.

|  | PEG-IFN/RBV  (n=18) | DCV/ASV  (n=18) | *P* value |
| --- | --- | --- | --- |
| Age at HCV eradication (years) | 68 (51-77) | 65 (49-76) | N.S. |
| Gender (male/female) | 7/11 | 8/10 | N.S. |
| Aspartate aminotransferase (IU/L) | 32 (15-337) | 35(13-272) | N.S. |
| Alanine aminotransferase (IU/L) | 34 (15-878) | 31(11-339) | N.S. |
| Albumin (g/dL) | 4.3 (3.5-4.9) | 4.2 (2.3-4.7) | N.S. |
| γ-glutamyl transpeptidase (IU/L) | 25 (14-285) | 37 (11-307) | N.S. |
| Platelet count (×10^4^/μL) | 15.9 (5.4-56.0) | 14.1 (6.8-25.6) | N.S. |
| Leukocyte count (×10^4^/μL) | 4530 (1810-8470) | 4240 (2090-8370) | N.S. |
| Hemoglobin (g/dL) | 13.7 (7.3-17.1) | 13.8 (9.9-16.8) | N.S. |
| Total cholesterol (mg/dL) | 183 (133-250) | 178(138-247) | N.S. |
| Triglyceride (mg/dL) | 99 (76-144) | 110 (54-212) | N.S. |
| HbA1c (%) | 5.2 (4.2-8.2) | 5.8 (4.6-8.6) | N.S. |
| Alfa-fetoprotein (ng/mL) | 5.6 (2.6-87.2) | 6.4 (3.1-207.1) | N.S. |
| Body mass index (kg/m^2^) | 22.3 (17.1-25.7) | 22.1 (15.8-30.4) | N.S. |
| Alcohol intake (yes/no) | 3/15 | 2/16 | N.S. |
| Hypertension (yes/no) | 5/13 | 7/11 | N.S. |
| Diabetes mellitus (yes/no) | 4/12 | 3/15 | N.S. |
| Hyperlipidemia (yes/no) | 3/15 | 4/14 | N.S. |
| FIB4 index (<3.25/3.25≤) | 10/8 | 11/7 | N.S. |
| FIB4 index | 2.7(0.99-10.541) | 2.8(0.75-10.889) | N.S. |
| HCV RNA (log/IU/mL) | 6.5 (3.7-7.3) | 6.4 (3.1-7.1) | N.S. |
| *IL28B* rs8099917 (TT/TG+GG) | 16/2 | 32/34 | N.S. |
| *DEPDC5* rs1012068 (TT/TG+GG) | 13/5 | 52/14 | N.S. |

Categorical data are represented as numbers of patients, and continuous data is represented as median and range.

PEG-IFN/RBV, peg-interferon plus ribavirin; DCV/ASV, daclatasvir plus; N.S., not significant; asunaprevir; alcohol intake, ≥80 g/day for more than 5 years
