# Supplementary material for: The risks of hepatocellular carcinoma development after HCV eradication are similar between patients treated with peg-interferon plus ribavirin and direct-acting antiviral therapy
Source: PLoS One. 2017 Aug 10;12(8):e0182710. doi: 10.1371/journal.pone.0182710 (PMC5552231; doi:10.1371/journal.pone.0182710)
Supplement: S2 Table — (DOCX) [file pone.0182710.s003.docx]

**Supporting table 2.** Characteristics of propensity score-matched patient treated with PEG-IFN plus RBV or daclatasvir plus asunaprevir between 2014 and 2015.

|  | PEG-IFN/RBV  (n=61) | DCV/ASV  (n=61) | *P* value |
| --- | --- | --- | --- |
| Age at HCV eradication (years) | 66 (40-76) | 67 (51-76) | N.S. |
| Gender (male/female) | 27/34 | 25/36 | N.S. |
| Aspartate aminotransferase (IU/L) | 39 (16-168) | 35(17-141) | N.S. |
| Alanine aminotransferase (IU/L) | 33(10-182) | 34(17-157) | N.S. |
| Albumin (g/dL) | 4.2 (2.3-5.3) | 4.2 (3.0-4.9) | N.S. |
| γ-glutamyl transpeptidase (IU/L) | 30 (12-449) | 27 (11-307) | N.S. |
| Platelet count (×10^4^/μL) | 13.9 (4.8-75.9) | 14.3 (5.6-41.2) | N.S. |
| Leukocyte count (×10^4^/μL) | 4620 (1810-9530) | 4420 (1380-8970) | N.S. |
| Hemoglobin (g/dL) | 13.4 (9.5-16.7) | 13.0 (8.5-16.2) | N.S. |
| Total cholesterol (mg/dL) | 169 (104-259) | 178(108-265) | N.S. |
| Triglyceride (mg/dL) | 99 (39-308) | 107 (41-517) | N.S. |
| HbA1c (%) | 5.2 (4.2-8.2) | 5.8 (4.2-8.6) | N.S. |
| Alfa-fetoprotein (ng/mL) | 6.4 (1.6-87.2) | 5.7 (1.2-116.4) | N.S. |
| Body mass index (kg/m^2^) | 22.7 (16.1-30.8) | 22.2 (17.7-30.2) | N.S. |
| Alcohol intake (yes/no) | 14/47 | 13/48 | N.S. |
| Hypertension (yes/no) | 22/39 | 30/31 | N.S. |
| Diabetes mellitus (yes/no) | 18/43 | 17/44 | N.S. |
| Hyperlipidemia (yes/no) | 18/43 | 23/38 | N.S. |
| FIB4 index (<3.25/3.25≤) | 27/34 | 17/44 | N.S. |
| FIB4 index | 2.9(0.329-13.727) | 2.8(0.436-10.385) | N.S. |
| HCV RNA (log/IU/mL) | 5.9 (1.3-7.3) | 6.1 (1.8-7.6) | N.S. |
| *IL28B* rs8099917 (TT/TG+GG) | 41/19 | 33/28 | N.S. |
| *DEPDC5* rs1012068 (TT/TG+GG) | 47/14 | 50/11 | N.S. |

Categorical data are represented as numbers of patients, and continuous data is represented as median and range.

PEG-IFN/RBV, peg-interferon plus ribavirin; DCV/ASV, daclatasvir plus; N.S., not significant; asunaprevir; alcohol intake, ≥80 g/day for more than 5 years
